# Chaos-embedded particle swarm optimization approach for protein-ligand docking and virtual screening

**DOI:** 10.1186/s13321-018-0320-9

**Published:** 2018-12-14

**Authors:** Hio Kuan Tai, Siti Azma Jusoh, Shirley W. I. Siu

**Affiliations:** 1 Department of Computer and Information Science, University of Macau, Avenida da Universidade, Taipa, Macau China; 2 Bioinformatics Lab, Faculty of Pharmacy, Level 8, FF2 Building, Universiti Teknologi MARA (UiTM), 42300 Bandar Puncak Alam, Selangor Malaysia

**Keywords:** Docking, Virtual screening, PSOVina, Autodock Vina, Chaotic maps, Singer map, Sinusoidal map

## Abstract

**Background:**

Protein-ligand docking programs are routinely used in structure-based drug design to find the optimal binding pose of a ligand in the protein’s active site. These programs are also used to identify potential drug candidates by ranking large sets of compounds. As more accurate and efficient docking programs are always desirable, constant efforts focus on developing better docking algorithms or improving the scoring function. Recently, chaotic maps have emerged as a promising approach to improve the search behavior of optimization algorithms in terms of search diversity and convergence speed. However, their effectiveness on docking applications has not been explored. Herein, we integrated five popular chaotic maps—logistic, Singer, sinusoidal, tent, and Zaslavskii maps—into PSOVina$^{{\mathrm{2LS}}}$, a recent variant of the popular AutoDock Vina program with enhanced global and local search capabilities, and evaluated their performances in ligand pose prediction and virtual screening using four docking benchmark datasets and two virtual screening datasets.

**Results:**

Pose prediction experiments indicate that chaos-embedded algorithms outperform AutoDock Vina and PSOVina in ligand pose RMSD, success rate, and run time. In virtual screening experiments, Singer map-embedded PSOVina$^{{\mathrm{2LS}}}$ achieved a very significant five- to sixfold speedup with comparable screening performances to AutoDock Vina in terms of area under the receiver operating characteristic curve and enrichment factor. Therefore, our results suggest that chaos-embedded PSOVina methods might be a better option than AutoDock Vina for docking and virtual screening tasks. The success of chaotic maps in protein-ligand docking reveals their potential for improving optimization algorithms in other search problems, such as protein structure prediction and folding. The Singer map-embedded PSOVina$^{{\mathrm{2LS}}}$ which is named PSOVina-2.0 and all testing datasets are publicly available on https://cbbio.cis.umac.mo/software/psovina.

**Electronic supplementary material:**

The online version of this article (10.1186/s13321-018-0320-9) contains supplementary material, which is available to authorized users.

## Introduction

Small-molecule drugs exert their pharmacological effects through binding to their biological targets and subsequently modulating the activities that are associated with diseases to be treated. To rationally design new drugs for a target protein, specific interactions of the binding partners must be correctly predicted. This prediction can be achieved with a computational approach called protein-ligand docking [1]. Given the three-dimensional structures of a target protein and a ligand, the main goal of protein-ligand docking is to dock the ligand at the active site of the protein and to score the different binding poses of the ligand. Then, through a virtual screening process, a large library of ligands can be docked, ranked and filtered according to their docking scores, enabling the rapid identification of lead candidates. Therefore, accurate and efficient docking tools are indispensable for reducing the cost and time in the drug discovery process.

From an algorithmic point of view, the docking problem is a conformational search problem, which is to find the combination of parameters that yields the optimal ligand binding pose. Assuming fixed topologies of the protein and ligand, then the conformational parameters of the complex include the position and orientation of the ligand with respect to the protein and the angles of all rotatable bonds of the ligand (and even the protein if flexibility of the protein is considered). Assessment of a ligand binding pose is done by a scoring function of interaction types and distances of the atoms between the protein and ligand, which can be force field-based, empirical-based or knowledge-based [2].

Various optimization strategies have been proposed to solve the protein-ligand docking problem. For example, a Monte Carlo (MC)-based approach was implemented in AutoDock Vina [3], and a genetic algorithm (GA)-based approach was implemented in GOLD [4] and AutoDock [5]. Recently, swarm-intelligence-based approaches using particle swarm optimization (PSO) and other nature-inspired methods, such as artificial bee colony (ABC) and ant colony optimization (ACO), have become very popular for solving nonlinear and complex optimization problems. The advantages of these metaheuristic algorithms are that they tend to find good solutions quickly, they are easy to implement, and there are many variants to allow easy customization of the algorithm fitting the domain of interest. Some metaheuristic docking methods have been implemented, such as SODOCK [6], PSO@AutoDock [7], FIPSDock [8], PSOVina [9] based on the PSO algorithm and variants, PLANTS [10] based on ACO and F*l*ABCps [11] based on ABC. All of these docking methods have been shown to improve the pose prediction accuracy and docking efficiency compared to traditional optimization methods. In these implementations, the metaheuristic algorithms were utilized as the global optimizer to quickly locate promising regions in the conformational search space. Some of these methods included a local search algorithm to refine the solution from the global search to the closest local minimum.

In this paper, we present an improvement of the PSOVina docking method that was previously developed in our group [9]. The first version of PSOVina implemented the canonical PSO algorithm with a convergence detection strategy to effectively reduce the execution time of AutoDock Vina docking by 51–60% [9]. The second version of PSOVina, named PSOVina$^{{\mathrm{2LS}}}$ [12], further enhanced the docking performance by incorporating a novel two-stage local search (2LS) algorithm to quickly examine the potential of the global search solutions. Only promising solutions will be refined by the expensive full-length local search. Our experimental results showed that the 2LS achieved an approximate threefold acceleration in finding optimal docking solutions relative to the conventional one-stage local search. In this work, we investigate the use of chaotic maps in PSOVina in an attempt to further enhance the search capability of the algorithm. A chaotic map is a function to mimic the dynamics of some nonlinear systems. Previous studies [13–15] indicated that using chaotic variables rather than the conventional random number generators might improve the search behavior of evolutionary algorithms in terms of search diversity and convergence speed. To evaluate the effectiveness of chaotic functions in docking applications, we implemented five chaotic functions in PSOVina$^{{\mathrm{2LS}}}$ and analyzed their performances on four benchmark docking datasets and two virtual screening datasets. In our experiments, chaos-embedded methods outperformed AutoDock Vina and our previous PSOVina in both the success rate of ligand pose prediction and the speed of virtual screening.

## Methods

### PSOVina and PSOVina$^{{\mathrm{2LS}}}$

PSOVina is a metaheuristic molecular docking program based on the AutoDock Vina software [3]. In PSOVina, the fast converging PSO algorithm was used as the global optimizer integrated with the Broyden–Fletcher–Goldfarb–Shanno (BFGS) local search algorithm and the scoring function of Vina. PSO is a population-based search method that is inspired by the social learning behaviors of bird flocking and fish schooling when searching for food [16]. The population, called *swarm*, consists of *N* members, called *particles*. Each individual particle represents a potential solution and moves in a *D*-dimensional search space based on its current position and velocity. During the search process, each particle adjusts its position according to its own experience and the swarm’s experience. The former is the best position that the particle has ever visited, called *pbest*, and the latter is the best position that the swarm has ever visited, called *gbest*. The velocity $V_i$ and position $X_i$ of the particle *i* are updated iteratively over time *t* according to the following equations:

1 $$\begin{aligned} V_i(t+1)&= w V_i(t)+c_1r_1(\text {pbest}_i(t)-X_i(t))\nonumber \\&\quad+ c_2r_2(\text {gbest}(t)-X_i(t)){,} \end{aligned}$$

2 $$\begin{aligned} X_i(t+1)&= X_i(t)+V_i(t+1){,} \end{aligned}$$

where $V_i = [v_{i1}, \ldots , v_{iD}]$ and $X_i = [x_{i1}, \ldots , x_{iD}]$. *w* is a constant parameter called the *inertia weight*, and it determines the contribution of the current velocity of the particle to its new velocity. A large *w* encourages exploration of the entire search space, and a small *w* facilitates local exploitation and convergence. Therefore, a suitable value of *w* (typically between 0.8 and 1.2) will help maintain a proper balance between the global and local search capabilities of the swarm. Rather than using a predefined constant value, many studies have proposed strategies to dynamically adjust *w* during the search process [17]. Two other coefficients $c_1$ and $c_2$ are the cognitive and social parameters, respectively. The former controls the particle’s movement toward the region where the best solution has been encountered by itself before, and the latter controls its movement toward the best region that the swarm has collectively found thus far. A similar treatment to adaptively adjust $c_1$ and $c_2$ was also proposed [18]. Finally, $r_1$ and $r_2$ are two uniform random variables between 0 and 1.

Although PSOVina has been improved in terms of global search efficiency, we found that each update step was still computationally expensive. The reason was that each particle update will undergo a local search step based on the BFGS algorithm to refine the solution to a local minimum. Since not all new solutions after the position update step of Eq. 2 are good solutions, we introduced the 2LS method into PSOVina to focus the computing resources on optimizing only promising solutions [12]. The first stage is to perform a short local search and decide the potential of a solution by comparing it to the current *pbest* solution obtained from a short local search. Only if the solution has improved energy will it enter the second stage to perform a complete local search for full optimization. This improved method, called PSOVina$^{{\mathrm{2LS}}}$, yielded greater prediction accuracy and achieved at least a threefold acceleration in run time compared to AutoDock Vina.

### Chaotic maps

Chaos is a bounded unstable dynamic behavior, and it has characteristics of randomness, ergodicity, initial value sensitivity, and regularity [19]. A chaotic sequence $\{x_n : n=0,1,2,\ldots \}$ is generated deterministically from a dynamical system of the form:

3 $$\begin{aligned} x_{n+1} = f(x_n), \quad n=0, 1, 2, \ldots \end{aligned}$$

where *f* is a smooth nonlinear function. Generation of the sequence is fast and depends on only one ($x_0$) or a few initial parameters, making it easy to use and store. Apart from regularity, an important difference between random sequences and random-like chaotic sequences is their assurance of ergodicity. Under proper conditions, they can cover all values without repeat within a certain range. The sequence generation is also very sensitive to the initial parameters; thus, only a slight modification of the parameters will produce an entirely different sequence.

Chaotic maps have been found to be promising for enhancing search performance in various optimization algorithms [13, 20, 21]. Generally, the selected chaotic function is used as a replacement of the conventional random number generator (mostly with a uniform distribution) or to adaptively modify parameters in the metaheuristic optimization algorithm while the search evolves. In this way, it is to prevent the search from becoming trapped in local optima and intends to improve the balance between exploring the search space and exploiting the found solutions. Successes have been reported in applying chaotic optimization in machine learning tasks such as feature selection and clustering [22, 23] and in real-world applications in the fields of engineering [24, 25], image processing [26], and recently in bioinformatics [27–29]. Well-known chaotic maps for these applications include logistic map [30], Singer map [31], sinusoidal map [31], tent map [32], Zaslavskii map [33], Gauss map [31], circle map [34], Arnold’s cat map [35], Sinai map [36], and piecewise map. Each of these maps are different with respect to the density of the periodic orbits and ways of mixing topologies. In the following, we introduce the characteristics of the five most used chaotic maps.

The logistic map is one of the simplest and most popular maps describing the nonlinear dynamics of a biological population [30]. This map is defined by the following equation:

4 $$\begin{aligned} x_{n+1}=\alpha x_n(1-x_n), \end{aligned}$$

where $\alpha$ is the control parameter. As shown in Additional file 1: Fig. S1, the dynamical behaviors in the logistic map systems will be either in the periodic regimes or in the chaotic regimes depending on the value of $\alpha$. In the former, only a finite set of different values will be visited, whereas in the latter, the system evolves in a disordered way and never repeats itself exactly. When $\alpha = 4$, $x_0\in (0,1)$ and $x_0\notin \{0.25, 0.5, 0.75\}$, this function generates $x_n$ covering the entire range of (0, 1).

The Singer map [31] is defined by the following equation:

5 $$\begin{aligned} x_{n+1} & = \mu (7.86 x_n - 23.31 x_n^2 + 28.75 x_n^3\nonumber \\&\quad- 13.302875 x_n^4), \end{aligned}$$

with $x_n\in (0,1)$ under the condition that $x_0\in (0,1)$ and $\mu \in [0.9, 1.08]$. Similar to the logistic map, systems with larger $\mu$ values evolve in the chaotic regimes, as shown in Additional file 1: Fig. S2.

The sinusoidal map [31] is defined by the following equation:

6 $$\begin{aligned} x_{n+1}=\alpha x_n^2 sin(\pi x_n){.} \end{aligned}$$

The systems clearly evolve in the chaotic regimes when $\alpha = 2.3$ and $0.45 \le x_0 \le 0.92$, as shown in Additional file 1: Fig. S3. Note that under these conditions, the chaotic values are $> 0.4$; thus, the generated states do not cover the entire range of (0, 1).

The tent map [32] and logistic map are topologically conjugate, and they have similar dynamical behaviors. The tent map is defined by the following equation:

7 $$\begin{aligned} x_{n+1}={\left\{ \begin{array}{ll} \mu x_n & x_n < 0.5{,} \\ \mu (1-x_n) & x_n \ge 0.5{,} \\ \end{array}\right. } \end{aligned}$$

where $\mu$ is a positive real constant. For optimization tasks, the following equation is mostly used  [13, 21, 23]:

8 $$\begin{aligned} x_{n+1}={\left\{ \begin{array}{ll} \frac{1}{0.7}x_n & x_n < 0.7{,} \\ \frac{10}{3}(1-x_n) & x_n \ge 0.7{.} \\ \end{array}\right. } \end{aligned}$$

The dynamical behavior of this system is shown in Additional file 1: Fig. S4.

The Zaslavskii map [33] is defined by the following equation:

9 $$\begin{aligned} x_{n+1} & = \left( x_n+v+ay_{k+1}\right) mod(1){,} \end{aligned}$$

10 $$\begin{aligned} y_{n+1} & = cos(2\pi x_n) + e^{-r}y_{n}{.} \end{aligned}$$

When $v=400$, $r=3$, $a=12$, and $y_{n+1}\in [-1.0512, 1.0512 ]$, the dynamical behaviors of Zaslavskii map systems are in a wide spectrum and very unpredictable, as shown in Additional file 1: Fig. S5.

### Chaos-embedded PSOVina

One of the main disadvantages of PSO is that it often suffers from becoming trapped in local optima, particularly when dealing with functions that have multiple local extrema. The consequence is premature convergence leading to suboptimal solutions. Previous studies on improving the global convergence of the PSO algorithm were largely focused on modifying the inertia weight *w* and acceleration coefficients $c_1$ and $c_2$ to prevent the swarm from becoming trapped in local optima. For example, Chuanwen and Bompard [24] used a logistic map in PSO to decide *w* iteratively based on the evolution number *t*. Their result showed that a chaos-embedded algorithm can achieve better performance in terms of efficiency and convergence rate. Recently, Alatas et al. [13] tested twelve chaos-embedded PSO methods and eight chaotic maps with different combinations of chaos-adapted sequences for coefficients *w*, $c_1$ and $c_2$ and chaos-adapted sequences for random variables $r_1$ and $r_2$. They concluded that chaos-adapted *w*, $r_1$ and $r_2$ performed the best in experiments on three benchmark mathematical functions. Both studies suggested that the use of chaotic maps as a replacement for the static parameters or the normal random sequences can improve the global search capability by more easily escaping the local minimum. In addition, it was hypothesized that chaotic maps add the ergodicity property in the search, which is lacking in random sequences [13].

To investigate the use of chaotic maps in protein-ligand docking, we embedded a chaotic map in PSOVina$^{{\mathrm{2LS}}}$ as a means to generate random numbers. The inertia weight constant (*w*) and random variables ($r_1$ and $r_2$) in the velocity update equation of Eq. 1 were replaced by chaotic variables:

11 $$\begin{aligned} V_i(t+1) & = w_{cm} V_i(t)+c_1r_{cm}(\text {pbest}_i(t)-X_i(t))\nonumber \\&\quad +c_2(1-r_{cm})(\text {gbest}(t)-X_i(t)){,} \end{aligned}$$

where $w_{cm}$ and $r_{cm}$ are variables of two independent chaotic sequences; both of these variables were initialized with different random values and updated before the velocity update step was performed. A few alternatives of when to iterate the chaotic function and the number of chaotic variables used were tested. It was found that when the chaotic variables were updated once for each particle, the search could yield better solutions. In total, we implemented five chaos-embedded PSOVina$^{{\mathrm{2LS}}}$ methods, including logistic, Singer, sinusoidal, tent, and Zaslavskii maps. Parameters of the chaotic maps used in the methods are listed in Table 1.

**Table 1:** The parameters of chaotic maps used in this study

Chaotic map	Parameter
Logistic map	$\alpha = 4$
Singer map	$\mu = 1.07$
Sinusoidal map	$\alpha = 2.3$
Zaslavskii map	$v=400$, $r=3$, $a=12$

### Datasets for pose prediction test

Four datasets were used to evaluate the ligand pose predictions of the docking methods: PDBbind, Astex, GOLD, and SB2010. PDBbind [37] is a manually curated database of 3D protein-ligand structures with experimentally obtained ligand binding affinities. We used the core-set complexes from PDBbind version 2014, which contains 195 protein-ligand complexes. These complexes were representatives of protein clusters generated from the PDBbind 3446 refined-set complexes. The selection was performed carefully to ensure that strong, medium, and weak binding cases were included in the core set. Both Astex [38] and GOLD [39] are widely used benchmark datasets for comparing docking methods. Astex contains 85 diverse protein-ligand structures with a resolution better than 2.5 Å. The set was derived from different drug discovery studies, and therefore, all ligands are drug-like samples. Among them, 23 of the ligands are approved drugs, and some are in clinical trials [38]. The GOLD dataset contains 77 protein-ligand complexes. This dataset was used for assessing state-of-the-art docking programs such as Surflex, Glide, MolDock and the more recent FIPSDock [38]. The fourth dataset is SB2012 from the Rizzo Lab [40]. It is an updated release of the SB2010 docking validation database containing a large dataset of 1043 crystallographic protein-ligand complexes. As a summary, information of the four datasets used for pose prediction tests are listed in Table 2.

All datasets were prepared by converting structure files into PDBQT format using the Python scripts prepare_receptor4.py and prepare_ligand4.py provided in the MGLTools package with the parameters ‘-A hydrogens’ and -U ‘nphs_lps_waters’. With these options, missing hydrogens were added, but nonpolar hydrogens were merged to the neighboring carbon based on the united-atom model scheme. As the ligand is treated as flexible in docking, this preprocessing step establishes torsion tree of the ligand that contains a fixed set of atoms (the root) and rotatable groups of atoms (the branches). All non-ring torsions are considered rotatable except bonds that only rotate hydrogens. For both protein and ligand, the default AD4 atom type and Gasteiger partial charges were used. Atoms such as *Au* and *Ce* that cannot be recognized by the conversion tools were removed. For each PDBbind receptor, the docking box was calculated based on the pocket residues given in the dataset for each complex, i.e., the geometrical center of all pocket atoms as the center of the box and the largest distances between pocket atoms in the X-, Y-, Z-dimensions as the box lengths. For the Astex diverse set, the prepared PDBQT files were kindly provided by the author of rDock [41]. For the GOLD benchmark set, the coordinates of the protein and ligand atoms were extracted from the PDB files, which were downloaded from RCSB PDB, whereas for the SB2012 docking validation set, structure files in MOL2 format were obtained from the Rizzo Lab page. Following the procedure in Ref. [8, 11], a default docking box size of 22.5 Å  in all three dimensions was created for receptors in the Astex, GOLD, and SB2012 datasets. The center of the docking box was defined as the geometric center of the bound ligand in the crystal structure.

**Table 2:** Four datasets for the pose prediction test

Name	Description	Number of complexes	References
PDBbind v2014 (core-set)	Representatives of protein clusters of high-quality structures selected from Protein Data Bank	195	[37]
Astex diverse set	Proteins are pharmaceutical or agrochemical targets; ligands are approved drugs or in clinical trials	85	[38]
GOLD benchmark set	Selected diverse complexes which were checked to be free from structural errors	77	[39]
SB2012 docking validation set	Ligands with a wide range of flexibilities	1043	[40]

### Datasets for virtual screening test

The Database of Useful Decoys-Enhanced (DUD-E) [42] was used in the virtual screening experiments. The entire dataset consists of 102 protein targets with known active ligands and computationally generated inactive ligands. The inactive ligands, called *decoys*, were made to have similar physicochemical properties such as molecular weight, number of rotatable bonds, calculated log P, and hydrogen bond acceptors and donors, but dissimilar 2D topologies from the active ligands such that it is challenging for docking programs to identify real positives from the positive-like ligands. Smaller subsets of four protein classes (G protein-coupled receptors, kinases, nuclear receptors, and proteases) are also available in DUD-E for family-specific virtual screening sets. In this study, due to the limited computing power, a diverse subset that contains 8 representative targets from different protein families (herein named DIV8) was employed to assess the screening performance of different docking methods. In addition, the nuclear receptor subset (herein named NR11) was also evaluated as a comprehensive test of one of the major drug target classes.

The sets of actives and decoys were preprocessed using the LigPrep module of Schrödinger 2017-1. In LigPrep, each ligand was first generated from the given isomeric SMILES string, and it was subsequently subject to 6 steps of preprocessing: (1) add hydrogen atoms to make all hydrogen explicit; (2) remove unwanted atoms such as counter ions; (3) neutralize functional groups, if possible, by adding or removing protons; (4) find low-energy conformations of flexible ring systems in the ligand; (5) filter distorted conformations by performing energy minimization; and (6) generate energy-minimized structure by performing a series of Monte Carlo multiple minimum (MCMM) searches. Finally, only one structure coordinate was retained. For the target receptor structures, they were preprocessed using the Protein Preparation Wizard of Schrödinger. It includes checking the structure for correct bond orders and correct protonation states (at pH 7.0), deleting far waters, optimizing the hydrogen bonding network, and performing energy minimization using the OPLS2005 force field. The optimized structures were then converted into PDBQT format using the prepare_ligand4.py and prepare_receptor4.py programs without any additional parameters; this ensures the programs that no repairs on the structures were required. For each receptor, size of the docking box was determined based on the co-crystallized ligand using the eBoxSize script. As shown in Ref. [43], eBoxSize can improve ranking accuracy in virtual screening experiments for about two-third of target proteins. The final virtual screening datasets with the numbers of generated actives and decoys are listed in Table 3.

**Table 3:** Number of actives and decoys in the DUD-E datasets for virtual screening test after preprocessing

Target	Type	Active	Decoy	Failed active$^{\mathrm{a}}$	Failed decoy$^{\mathrm{a}}$
(a) DIV8: Diverse target subset					
akt1	Kinase	293	16,448	0	2
ampc	Enzyme	48	2850	0	0
cp3a4	Cytochrome	170	11,798	0	2
cxcr4	G protein-coupled receptor	40	3414	0	0
gcr	Nuclear receptor	258	14,996	0	4
hivpr	Protease	536	35,743	0	7
hivrt	Enzyme	337	18,887	1	4
kif11	Other	116	6847	0	3
Total		1794	110,983	1	22

Target	Type	Active	Decoy	Failed active$^{\mathrm{a}}$	Failed decoy$^{\mathrm{a}}$
(b) NR11: Nuclear receptor target subset					
andr	Androgen receptor	269	14,349	0	1
esr1	Estrogen receptor alpha	383	20,685	0	0
esr2	Estrogen receptor beta	367	20,199	0	0
gcr	Glucocorticoid receptor	258	14,996	0	4
mcr	Mineralocorticoid receptor	94	5150	0	0
ppara	Peroxisome proliferator-activated receptor alpha	373	19,399	0	0
ppard	Peroxisome proliferator-activated receptor delta	240	12,250	0	0
pparg	Peroxisome proliferator-activated receptor gamma	484	25,298	0	2
prgr	Progesterone receptor	293	15,814	0	0
rxra	Retinoid X receptor alpha	131	6950	0	0
thb	Thyroid hormone receptor beta-1	103	7450	0	0
Total		2995	162,540	0	7

$^{\mathrm{a}}$Actives and decoys which failed to pass all the preprocessing steps were not included in the virtual screening experiments

### Performance analysis

When the structure of the co-crystallized ligand is given, the standard root-mean-square deviation (RMSD) can be used to evaluate the accuracy of the predicted ligand binding pose. RMSD is a measure of the difference between the predicted position of each ligand atom and its actual position in the experimental structure with respect to the target protein. In this work, a predicted ligand pose with an RMSD of 2 Å  or less was considered successful.

Being a stochastic algorithm, PSOVina (also AutoDock Vina) can provide different solutions in repeated runs. For pose prediction experiments, we performed docking of each complex 10 times and reported the performance measured from the best-scoring pose over all repeated runs (i.e., the docking pose found with the lowest binding affinity in 10 runs). We also measured the average performance of each run. For fairness, the same repetitive experiment was executed for all methods to be compared.

The performance of a docking method in virtual screening was evaluated based on the list of the screened compounds ranked by the predicted binding affinity. The more actives that are ranked high in the list, the more effective is the docking method for virtual screening. Two metrics were used in this study: the area under the receiver operating characteristic curve (AUC-ROC) and the enrichment factor (EF). The former is the global performance measure of a method from the ratios of true positive fraction over the false positive fraction at different classification thresholds. Ligands in the list above the threshold are classified as actives, whereas those below the threshold are classified as decoys. An AUC-ROC value of 1.0 indicates perfect classification, whereas a value of  0.5 indicates random prediction. Because drug discovery research will mainly consider the top-ranked ligands from the virtual screening result for further investigation, a measure of how good is the predicted top-$x\%$ ranked ligands is more indicative about the effectiveness of the docking method for virtual screening. The value of $\text {EF}_{x\%}$ is computed as:

12 $$\begin{aligned} \text {EF}_{x\%} = \frac{\text {actives at~} x\%}{\text {total actives}} \Bigg / \frac{\text {ligands at~} x\%}{\text {total ligands}}{.} \end{aligned}$$

Program efficiency, i.e., the run time, was measured as the elapsed time (or referred to as the **real** time) used by the docking program with the Linux command time.

Pose prediction tests were performed on a Dell XPS 8700 desktop with an Intel i7 quad-core 3.6 GHz processor and 24 GB of memory running Ubuntu 15. Virtual screening tests were run on a high-performance computing (HPC) cluster, where each node was equipped with a 24-core Intel Xeon E5-2690 GHz CPU and 256 GB of memory.

## Results and discussion

To evaluate the effectiveness of chaotic maps in protein-ligand docking, two types of experiments were performed: ligand pose prediction and virtual screening.

### Comparison of ligand pose prediction accuracy and docking speed

We conducted experiments using four independent datasets, namely, PDBbind, Astex, GOLD, and SB2012, to evaluate the docking performances of chaos-embedded PSOVina$^{{\mathrm{2LS}}}$ methods and compare them to AutoDock Vina and our previous versions of PSOVina. For PSOVina and PSOVina$^{{\mathrm{2LS}}}$, the following PSO parameters were used: $N=8$, $w=0.36$, and $c_1=c_2=0.99$. For PSOVina$^{{\mathrm{2LS}}}$, two additional parameters, $R=0.1$ and $C_r=18$, for the 2LS were used. For each complex in the dataset, 10 docking repetitions were performed, and the binding pose with the lowest binding affinity among the predicted poses was taken as the final docking solution.

The docking performances of five chaotic maps are compared in Table 4. Using the best-scoring pose among ten repeats as the final solution for each complex, the RMSDs and success rates of PSOVina and PSOVina$^{{\mathrm{2LS}}}$ are consistently better than those of AutoDock Vina. When a chaotic map was employed as a random number generator, variants of chaos-embedded PSOVina$^{{\mathrm{2LS}}}$ show a further improvement in success rate and in most cases also in RMSD. Regarding the average RMSD and success rate, there are more variations, presumably due to the stochastic nature of the docking algorithms. In all cases, PSOVina$^{{\mathrm{2LS}}}$ has the shortest run time. Replacing random numbers with chaotic sequences only introduced minor additional computing cost.

We summarize the overall pose prediction performances of the docking methods in Table 5. PSOVina$^{{\mathrm{2LS}}}$ with sinusoidal map yielded the highest best-scoring pose success rate of 74.62%, followed by Singer map with a rate of 73.21% and logistic map with a rate of 72.72%. AutoDock Vina only achieved a 65.68% success rate, and PSOVina$^{{\mathrm{2LS}}}$ achieved 70.89%. The fastest method is PSOVina$^{{\mathrm{2LS}}}$, which gained an almost sixfold acceleration with respect to AutoDock Vina, while chaos-embedded methods in general achieved a fivefold acceleration in docking.

Therefore, the experimental results presented in this section are strong evidence that chaotic maps can improve the global exploration capability of the PSO algorithm in protein-ligand docking and predict higher-quality docking poses than nonchaotic methods in a shorter amount of time. Specifically, sinusoidal map and Singer map appear to be the best options for the pose prediction considering the tradeoff between accuracy and run time.

**Table 4:** Docking performance comparison of AutoDock Vina, PSOVina, PSOVina$^{{\mathrm{2LS}}}$, and chaos-embedded PSOVina$^{{\mathrm{2LS}}}$ methods on four pose prediction datasets

	Best-scoring pose RMSD (Å)$^{\mathrm{a}}$	Average RMSD (Å)	Best-scoring pose success rate (%)$^{\mathrm{a}}$	Average success rate (%)	No. of iterations$^{\mathrm{b}}$	Run time (s)$^{\mathrm{b}}$
(a) PDBBind v.2014 dataset						
AutoDock Vina	2.68393	2.70336	62.56	61.33	22777	21.46
PSOVina	2.27188	2.50727	68.21	64.67	892	8.97
PSOVina$^{{\mathrm{2LS}}}$	2.14915	2.79023	70.77	61.03	957	3.43
Chaos-embedded PSOVina$^{2LS}$						
Logistic map	1.95241	2.61573	72.82	63.49	1053	3.75
Singer map	1.98661	2.52277	72.82	64.26	1069	3.75
Sinusoidal map	*1.90650*	2.73205	*74.36*	61.33	1105	3.82
Tent map	2.07797	2.77287	69.23	60.92	981	3.54
Zaslavskii map	1.98789	2.65951	72.31	62.00	1015	3.67
(b) Astex diverse dataset						
AutoDock Vina	1.90681	1.92633	71.76	71.53	20086	18.53
PSOVina	1.82160	1.71506	74.12	76.35	1392	8.21
PSOVina$^{{\mathrm{2LS}}}$	1.58374	1.87782	75.29	72.59	885	2.63
Chaos-embedded PSOVina$^{2LS}$						
Logistic map	1.63183	1.90169	76.47	71.65	951	2.82
Singer map	1.61686	1.88862	77.65	72.35	1097	3.05
Sinusoidal map	*1.50551*	1.99939	*80.00*	71.06	1234	3.30
Tent map	1.54835	1.91905	78.82	72.12	968	2.85
Zaslavskii map	1.54228	1.84950	78.82	72.12	928	2.72
(c) GOLD benchmark set						
AutoDock Vina	2.78586	2.91744	64.94	63.25	20071	19.91
PSOVina	2.59811	2.58979	66.23	66.75	1289	7.64
PSOVina$^{{\mathrm{2LS}}}$	2.41496	2.85823	71.43	60.91	897	2.75
Chaos-embedded PSOVina$^{2LS}$						
Logistic map	2.32352	2.71251	*75.32*	64.42	1002	2.97
Singer map	2.50710	2.73068	71.43	62.73	990	2.97
Sinusoidal map	2.27549	2.61833	74.03	64.81	1065	3.15
Tent map	*2.23369*	2.69675	70.13	62.60	916	2.72
Zaslavskii map	2.45169	2.80725	72.73	62.73	866	2.69
(d) SB2012 docking validation dataset						
AutoDock Vina	2.64185	2.77003	63.47	61.79	22977	20.33
PSOVina	2.38248	2.64763	65.68	62.78	1372	12.77
PSOVina$^{{\mathrm{2LS}}}$	2.29462	2.91399	66.06	58.12	1036	3.04
Chaos-embedded PSOVina$^{2LS}$						
Logistic map	2.41665	2.91596	66.25	57.94	1112	3.31
Singer map	*2.11773*	2.94298	*70.95*	57.48	1138	3.25
Sinusoidal map	2.16409	3.08916	70.09	54.67	1133	3.03
Tent map	2.17928	2.99936	69.22	56.74	1066	3.21
Zaslavskii map	2.35440	2.94977	66.06	57.17	1081	3.27

$^{\mathrm{a}}$ The best-scoring pose is the pose with the lowest binding affinity in docking repeats. Thus, best-scoring pose RMSD and success rate are the average RMSD and success rate of the best-scoring poses of all complexes in the dataset

$^{\mathrm{b}}$ No. of iterations and run time were averaged from all docking instances

Best results are shown in italics

**Table 5:** Overall pose prediction performance of AutoDock Vina, PSOVina, PSOVina$^{{\mathrm{2LS}}}$, chaos-embedded PSOVina$^{{\mathrm{2LS}}}$ methods

	Best-scoring pose RMSD (Å)	Best-scoring pose success rate (%)	Run time (s)
AutoDock Vina	2.50 (0.40)	65.68 (4.17)	17.56 (5.24)
PSOVina	2.27 (0.33)	68.56 (3.86)	9.40 (2.31)
PSOVina$^{{\mathrm{2LS}}}$	2.11 (0.37)	70.89 (3.79)	*2.96* (0.36)
Chaos-embedded PSOVina$^{2LS}$			
Logistic map	2.08 (0.36)	72.72 (4.57)	3.21 (0.41)
Singer map	2.06 (0.37)	73.21 (3.06)	3.26 (0.35)
Sinusoidal map	*1.96* (0.34)	*74.62* (4.08)	3.33 (0.35)
Tent map	2.01 (0.31)	71.85 (4.67)	3.08 (0.37)
Zaslavskii map	2.08 (0.41)	72.48 (5.21)	3.09 (0.47)

Best results are shown in italics

### Comparison of virtual screening accuracy and screening speed

To assess the screening performances of the chaos-embedded docking methods, we performed virtual screening experiments using the DUD-E diverse target subset (DIV8) and the nuclear receptor target subset (NR11). Four docking methods, namely, AutoDock Vina, PSOVina, Singer map-embedded PSOVina$^{{\mathrm{2LS}}}$, and sinusoidal map-embedded PSOVina$^{{\mathrm{2LS}}}$, were compared with respect to the values of AUC-ROC, EF, and run time. Only one docking was performed per complex. PSO parameters were the same as in the pose prediction experiments except that more particles were used ($N=16$).

**Fig. 1:**
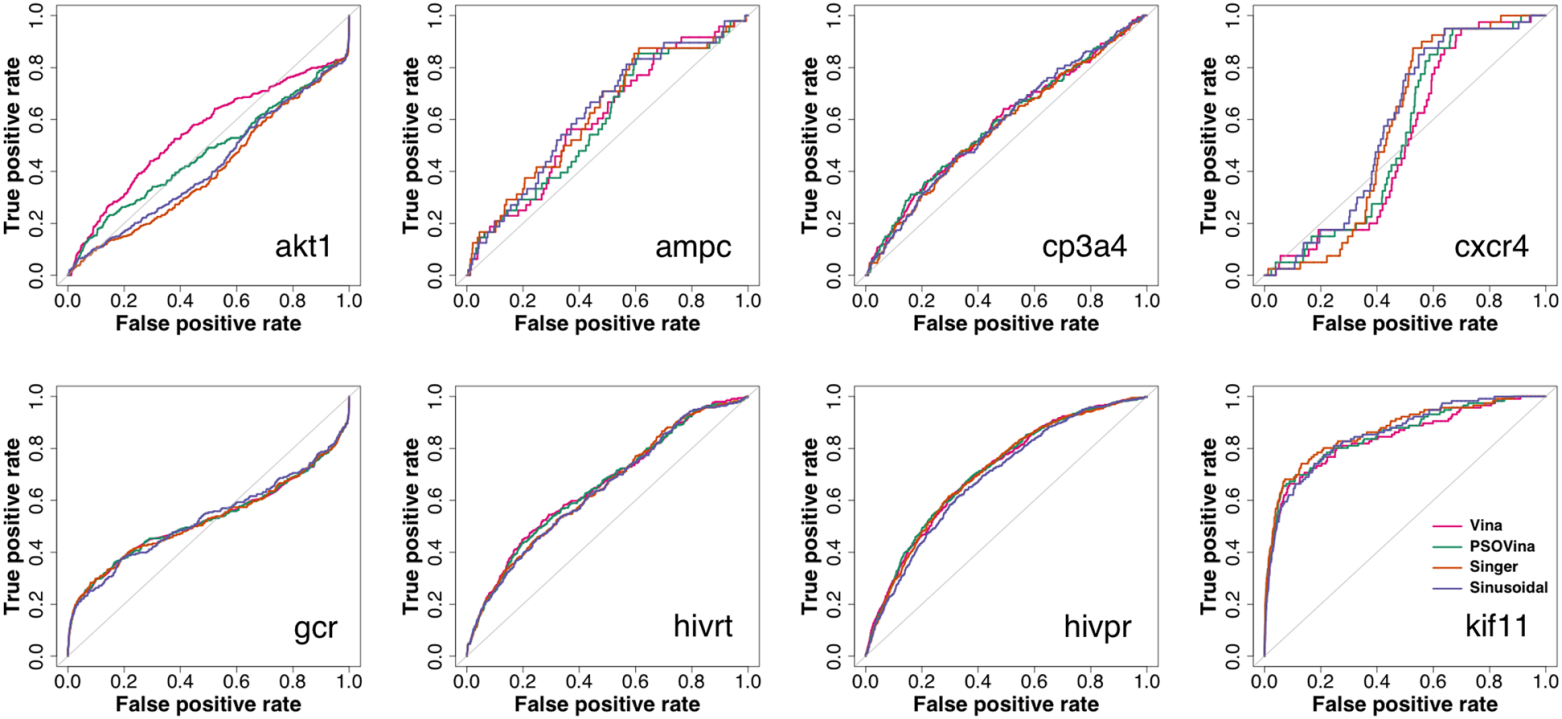
ROC curves of virtual screening the DUD-E diverse targets using AutoDock Vina, PSOVina, and chaos-embedded PSOVina$^{{\mathrm{2LS}}}$ with Singer and sinusoidal maps

**Fig. 2:**
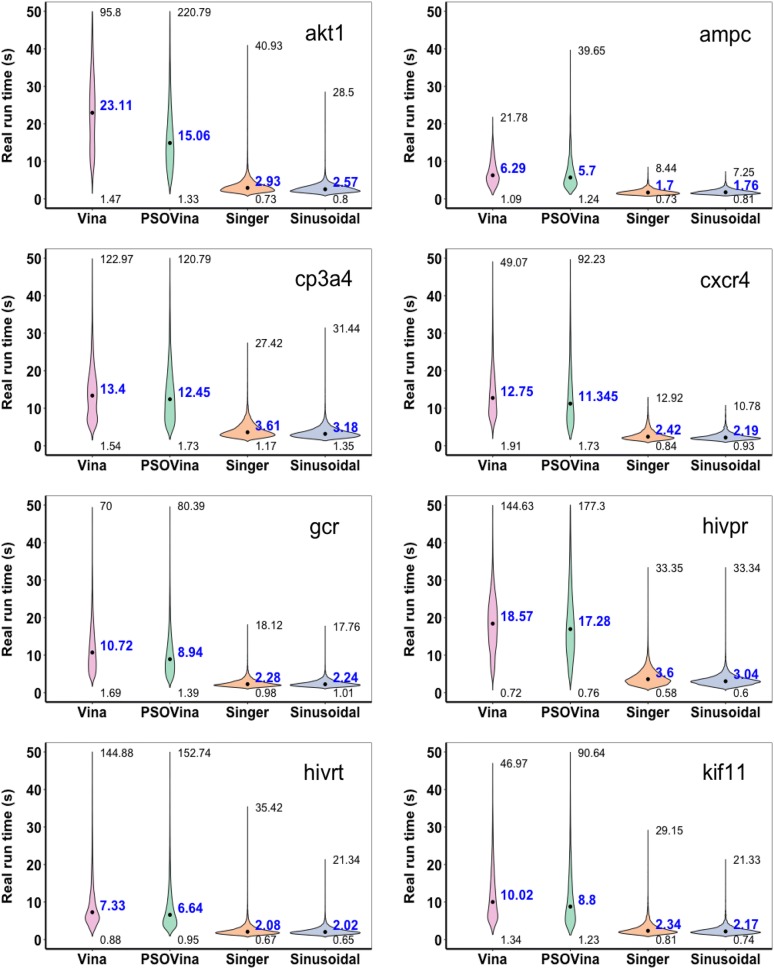
Run time (in seconds) of virtual screening the DUD-E diverse targets. Text annotations in the violin plot indicate the maximum (top), median (text in blue, location of the median shown as a black dot), and minimum (bottom) run times by each method

The DIV8 results are presented in Figs. 1 and  2 and Table 6, while the NR11 results are presented in Table 7 and Additional file 1: Figs. S6 and S7. The ROC curves show that all docking methods generated very similar ranking lists except for a few targets (DIV8’s akt1 and NR11’s ppara, ppard, and pparg). For DIV8, the AUC-ROCs averaged across targets are 0.62, 0.62, 0.61 and 0.62 for Vina, PSOVina, Singer, and sinusoidal, respectively. Singer has the largest mean EF_1%_ of 6.11, followed by PSOVina (5.92), sinusoidal (5.59) and finally Vina (5.38), whereas Autodock Vina has the largest mean EF_20%_ of 1.93, followed by PSOVina (1.91), sinusoidal (1.81) and Singer (1.77). However, these differences are statistically indistinguishable as suggested by paired Student’s *t*-test between pairs of the docking methods at the significance level of $\alpha =0.05$ (see Additional file 1: Table S1). Similarly, for NR11 the screening performance of Singer is comparable to AutoDock Vina and PSOVina in terms of AUC-ROC and EF_1%_. It performs only slightly worse than AutoDock Vina in EF_20%_ with a *p*-value of 0.04199. In contrast, sinusoidal performs slightly worse than AutoDock Vina in both AUC-ROC and EF_20%_ with *p*-values of 0.04996 and 0.04755. Therefore, in terms of screening accuracy Singer map-embedded PSOVina$^{{\mathrm{2LS}}}$ is preferable to sinusoidal-map embedded method.

**Fig. 3:**
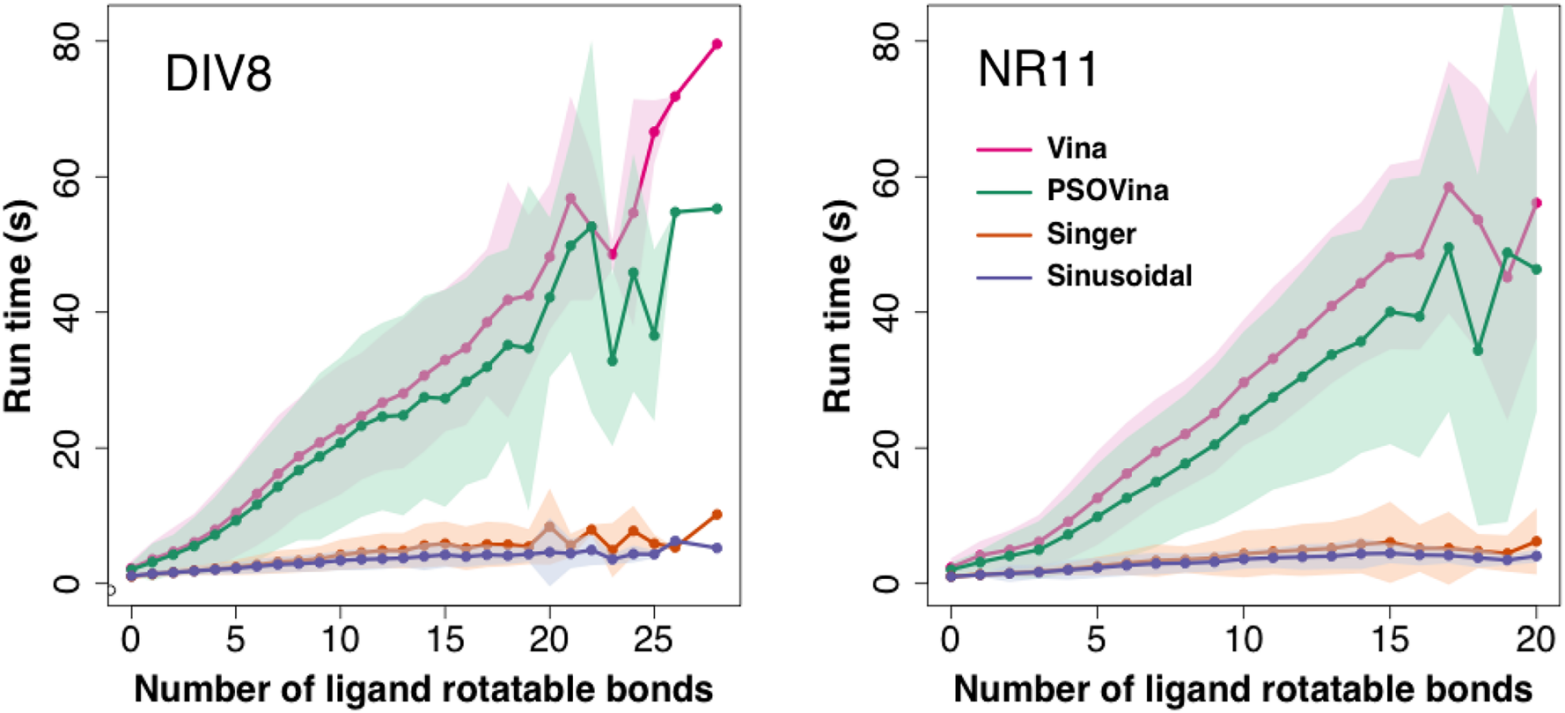
Average run time (in seconds) versus number of ligand rotatable bonds

After confirming the screening accuracies of choas-embedded methods, we evaluated their screening speed. Figure 2 and Additional file 1: Fig. S7 show the violin plots of run time used by different methods in screening all compounds in the DIV8 and NR11 datasets, respectively. Notably, while the median run times varied in a wide range of approximately 6–23 s for AutoDock Vina and 6–17 s for PSOVina, the chaos-embedded methods varied in a small range of only approximately 1–3 s in screening the DIV8 dataset. The same observation can be obtained from the run time analysis of the NR11 virtual screening experiments. Taken together two data sets, sinusoidal has the shortest average run time of 2.51 s, followed by Singer of 2.81 s, PSOVina of 12.06 s and AutoDock Vina of 14.93 s. As indicated by the paired Student’s *t*-test (see Additional file 1: Table S1), the speed improvements of the chaos-embedded methods over AutoDock Vina and PSOVina are very significant at $\alpha =0.01$, achieving an average of five- to sixfold acceleration, where sinusoidal seems slightly faster than Singer at $\alpha =0.05$. As the docking run time is proportional to the number of rotatable bonds of the ligand and the size of the receptor pocket, we further analyzed the median run time with respect to the number of ligand rotatable bonds using the DIV8 and NR11 datasets. As shown in Fig. 3 docking run time of chaos-embedded methods is only minimally affected by the increase of the number of torsions.

**Table 6:** Results of area under ROC curves (AUC-ROC) and enrichment factor (EF) of virtual screening the DUD-E diverse targets (DIV8) using AutoDock Vina, PSOVina, and chaos-embedded PSOVina$^{{\mathrm{2LS}}}$ with Singer and sinusoidal maps

Target	AutoDock Vina			PSOVina			Singer			Sinusoidal		
	AUC	EF$_{1\%}$	EF$_{20\%}$	AUC	EF$_{1\%}$	EF$_{20\%}$	AUC	EF$_{1\%}$	EF$_{20\%}$	AUC	EF$_{1\%}$	EF$_{20\%}$
akt1	0.55	0.00	1.52	0.47	1.71	1.31	0.40	1.37	0.75	0.42	2.05	0.84
ampc	0.60	0.00	1.25	0.59	0.00	1.46	0.62	2.08	1.56	0.63	2.08	1.56
cp3a4	0.58	0.60	1.65	0.59	1.19	1.62	0.57	1.19	1.53	0.58	1.79	1.53
cxcr4	0.52	0.00	0.87	0.54	0.00	0.75	0.59	0.00	0.25	0.59	0.00	0.87
gcr	0.53	10.43	1.98	0.53	10.82	1.88	0.53	11.59	1.90	0.53	11.98	1.88
hivpr	0.71	4.10	2.31	0.71	3.17	2.38	0.71	2.98	2.34	0.69	3.17	2.17
hivrt	0.66	4.77	2.20	0.65	4.77	2.17	0.65	4.77	1.93	0.64	4.77	1.92
kif11	0.84	23.15	3.66	0.85	25.73	3.71	0.87	24.87	3.92	0.86	18.87	3.71
Average	0.62	5.38	1.93	0.62	5.92	1.91	0.61	6.11	1.77	0.62	5.59	1.81

**Table 7:** Results of area under ROC curves (AUC-ROC) and enrichment factor (EF) of virtual screening the DUD-E nuclear receptor targets (NR11) using AutoDock Vina, PSOVina, and chaos-embedded PSOVina$^{{\mathrm{2LS}}}$ with Singer and sinusoidal maps

Target	AutoDock Vina			PSOVina			Singer			Sinusoidal		
	AUC	EF$_{1\%}$	EF$_{20\%}$	AUC	EF$_{1\%}$	EF$_{20\%}$	AUC	EF$_{1\%}$	EF$_{20\%}$	AUC	EF$_{1\%}$	EF$_{20\%}$
andr	0.57	11.54	1.93	0.57	11.17	1.90	0.57	11.17	1.84	0.57	10.79	1.88
esr1	0.75	13.82	2.74	0.74	13.56	2.60	0.74	11.73	2.62	0.73	12.77	2.69
esr2	0.77	11.70	3.09	0.76	10.88	3.04	0.76	12.51	3.00	0.76	13.06	3.05
gcr	0.53	10.43	1.98	0.53	10.82	1.88	0.53	11.59	1.90	0.53	11.98	1.88
mcr	0.53	3.22	1.54	0.53	3.22	1.54	0.53	3.22	1.60	0.53	3.22	1.54
ppara	0.85	4.55	3.65	0.80	2.68	2.98	0.75	2.14	2.60	0.73	1.07	2.24
ppard	0.81	2.50	3.33	0.79	2.08	2.75	0.72	0.83	2.06	0.74	1.67	2.17
pparg	0.79	5.57	3.11	0.76	3.30	2.52	0.72	2.89	2.13	0.70	2.68	2.02
prgr	0.61	9.56	2.25	0.61	9.56	2.24	0.61	9.56	2.22	0.60	9.90	2.25
rxra	0.83	33.50	3.55	0.83	32.74	3.47	0.81	33.50	3.51	0.81	32.73	3.44
thb	0.81	26.05	3.35	0.81	25.09	3.40	0.81	27.02	3.25	0.80	25.08	3.25
Average	0.71	12.04	2.78	0.70	11.37	2.57	0.69	11.47	2.43	0.68	11.36	2.40
Average$^{\mathrm{a}}$	0.67	14.98	2.55	0.67	14.63	2.51	0.67	15.04	2.49	0.67	14.94	2.50

$^{\mathrm{a}}$Averaged without ppara, ppard and pparg

## Conclusion

In this work, we explored the use of chaotic maps to enhance the search capability and speed in docking applications. Based on our previous version of PSOVina$^{{\mathrm{2LS}}}$, chaos-embedded docking algorithms of five popular chaotic maps were implemented. These algorithms were tested using four docking benchmark datasets for ligand pose prediction performance and two DUD-E subsets for virtual screening performance. The results of our analysis showed that chaos-embedded methods are superior in terms of ligand pose RMSD and docking success rate. In particular, Singer-embedded PSOVina$^{{\mathrm{2LS}}}$ gained a significant five- to sixfold acceleration in virtual screening experiments with similar screening accuracies to AutoDock Vina in terms of AUC-ROC and EF. Taken together, our results suggest that chaos-embedded PSOVina$^{{\mathrm{2LS}}}$ methods might be better alternatives than AutoDock Vina in virtual screening. The success of chaotic maps in protein-ligand docking reveals their potential for improving optimization algorithms in other molecular conformational search problems, such as protein structure prediction and folding.

## Additional file

**Additional file 1.**  Dynamical behaviors of chaotic maps, virtual screening results of the DUD-E NR11 subset, and statistical test results of virtual screening performances.
