# Supplementary material for: Chaos-embedded particle swarm optimization approach for protein-ligand docking and virtual screening
Source: J Cheminform. 2018 Dec 14;10:62. doi: 10.1186/s13321-018-0320-9 (PMC6755579; doi:10.1186/s13321-018-0320-9)
Supplement: Supplementary file 1 — Additional file 1. Dynamical behaviors of chaotic maps, virtual screening results of the DUD-E NR11 subset, and statistical test results of virtual screening performances. [file 13321_2018_320_MOESM1_ESM.pdf]

Supplementary Information

Chaos-embedded particle swarm optimization approach for protein-ligand docking and virtual screening

Hio Kuan Tai,[†] Siti Azma Jusoh,[‡] and Shirley W. I. Siu[*,†]

†*Department of Computer and Information Science, University of Macau, Macau*

‡*Faculty of Pharmacy, Universiti Teknologi MARA, Malaysia*

E-mail: shirleysiu@umac.mo

Phone: +853 8822 4452. Fax: +853 8822 2426

Content:

I. Dynamical behaviors of chaotic maps

II. Virtual screening results of the DUD-E nuclear receptor subset

III. Statistical test results of virtual screening performances

I. Dynamical behaviors of chaotic maps

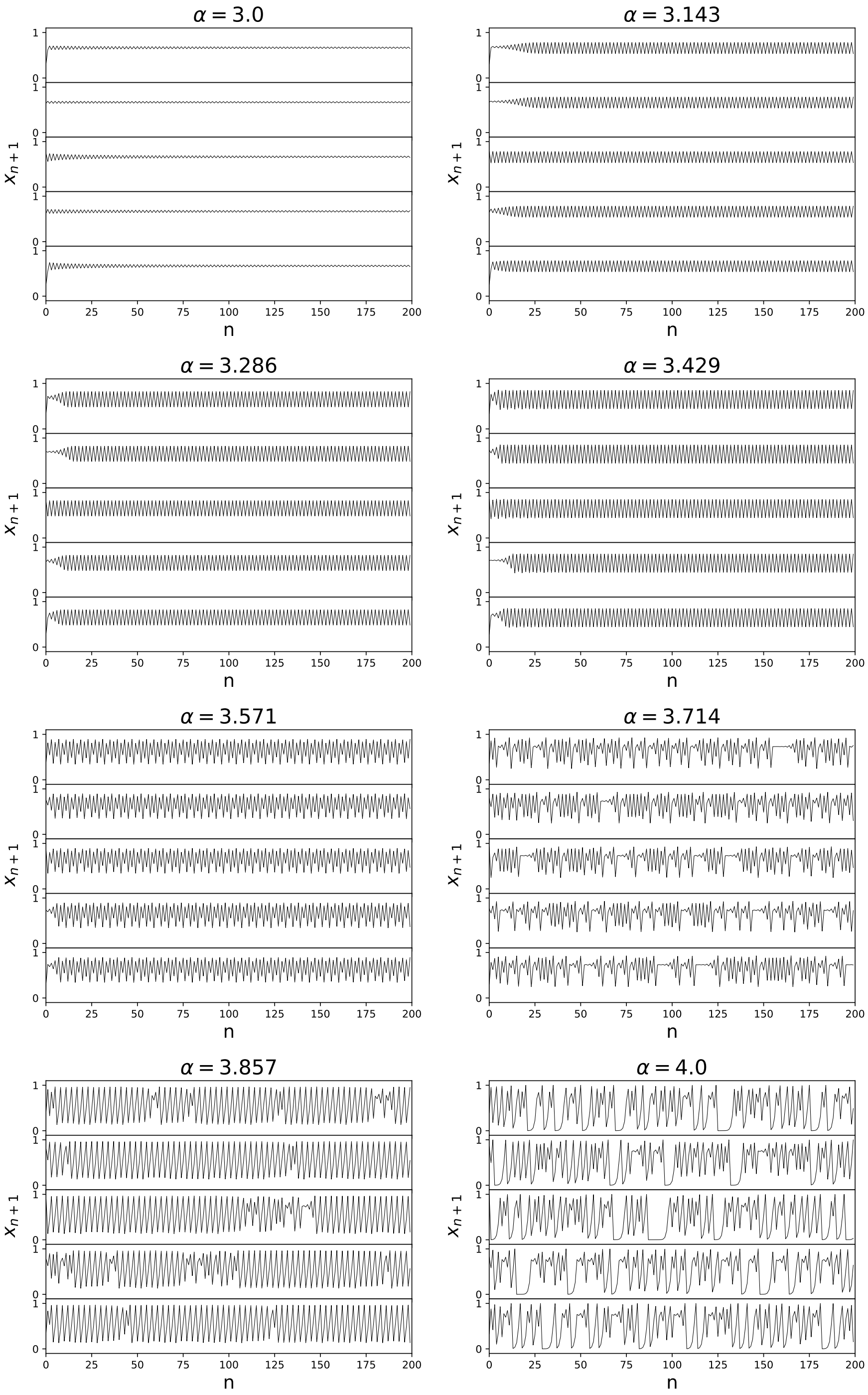

Figure S1: The dynamical behaviors in the logistic map systems with different α values. Each subplot shows 5 chaotic sequences (top to bottom) with $x_0 = 0.11, 0.31, 0.51, 0.71$, and 0.91, respectively.

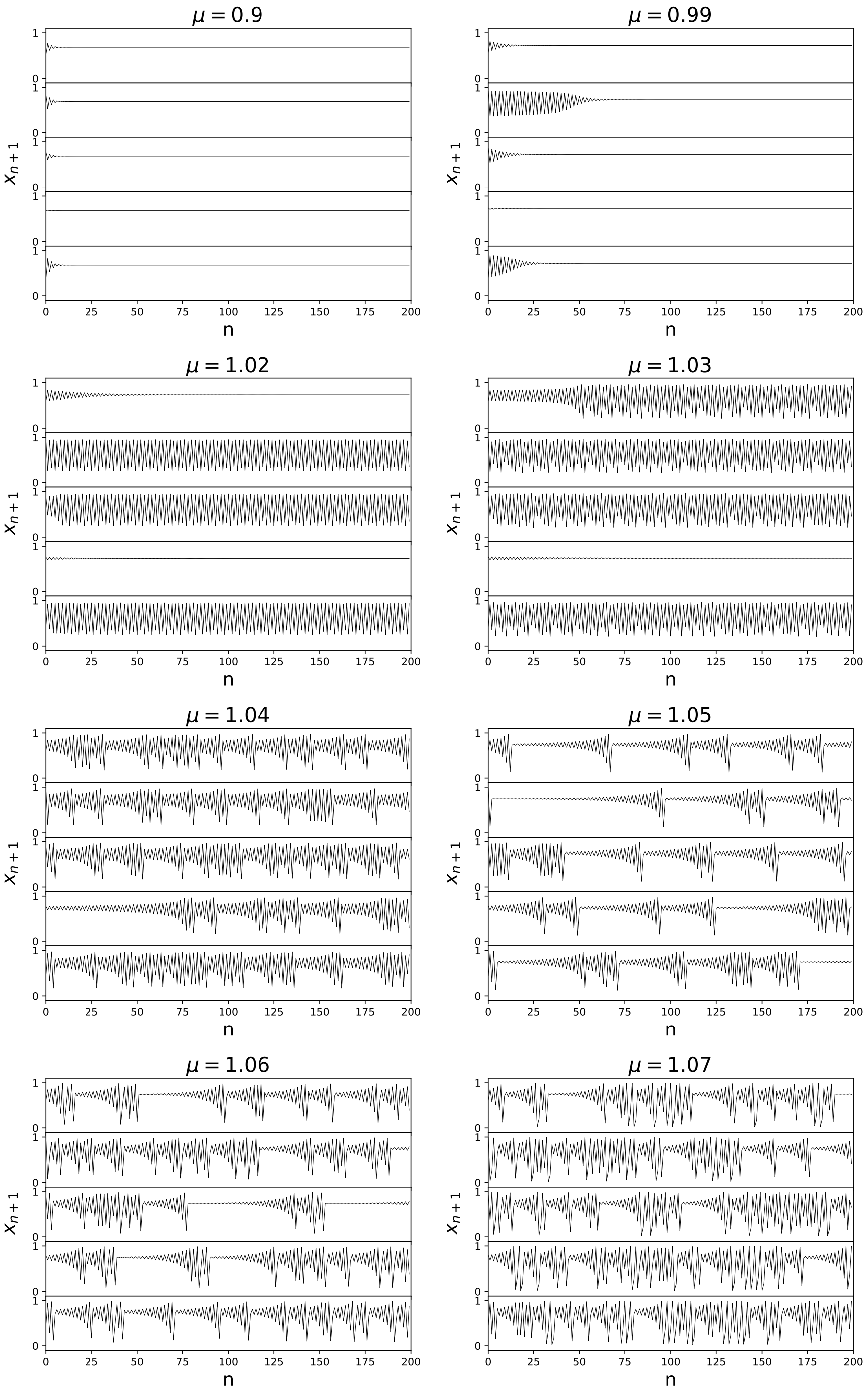

Figure S2: The dynamical behaviors in the Singer map systems with different μ values. Each subplot shows 5 chaotic sequences (top to bottom) with $x_0 = 0.1, 0.3, 0.5, 0.7,$ and 0.9, respectively.

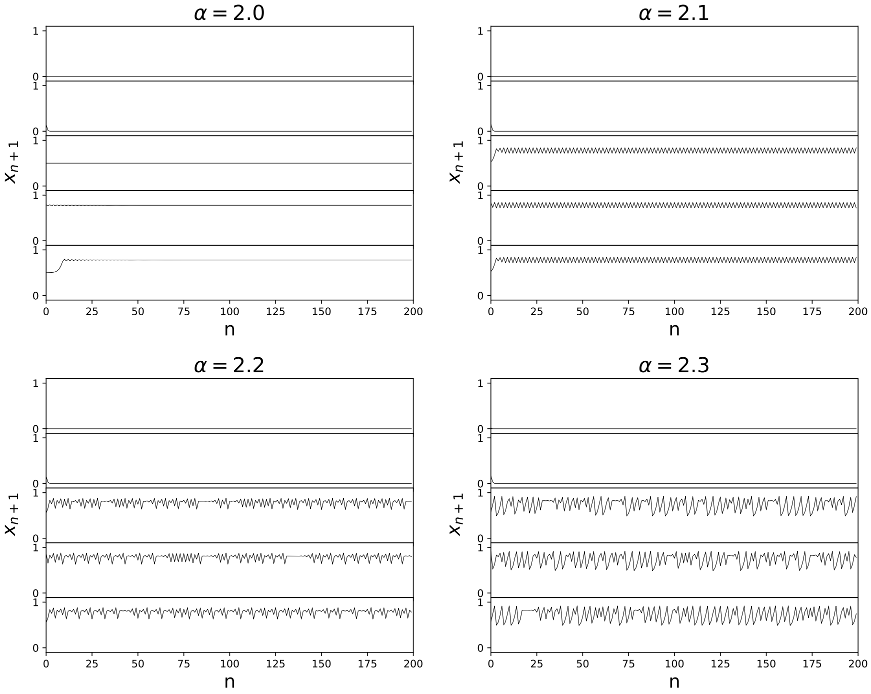

Figure S3: The dynamical behaviors in the sinusoidal map systems with different α values. Each subplot shows 5 chaotic sequences (top to bottom) with $x_0 = 0.1, 0.3, 0.5, 0.7$, and 0.9, respectively.

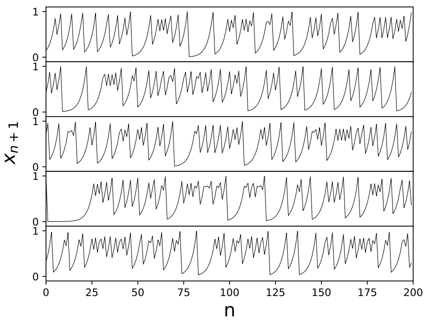

Figure S4: The dynamical behavior in the tent map system. The subplot shows 5 sequences (top to bottom) with $x_0 = 0.1, 0.3, 0.5, 0.7$, and 0.9, respectively.

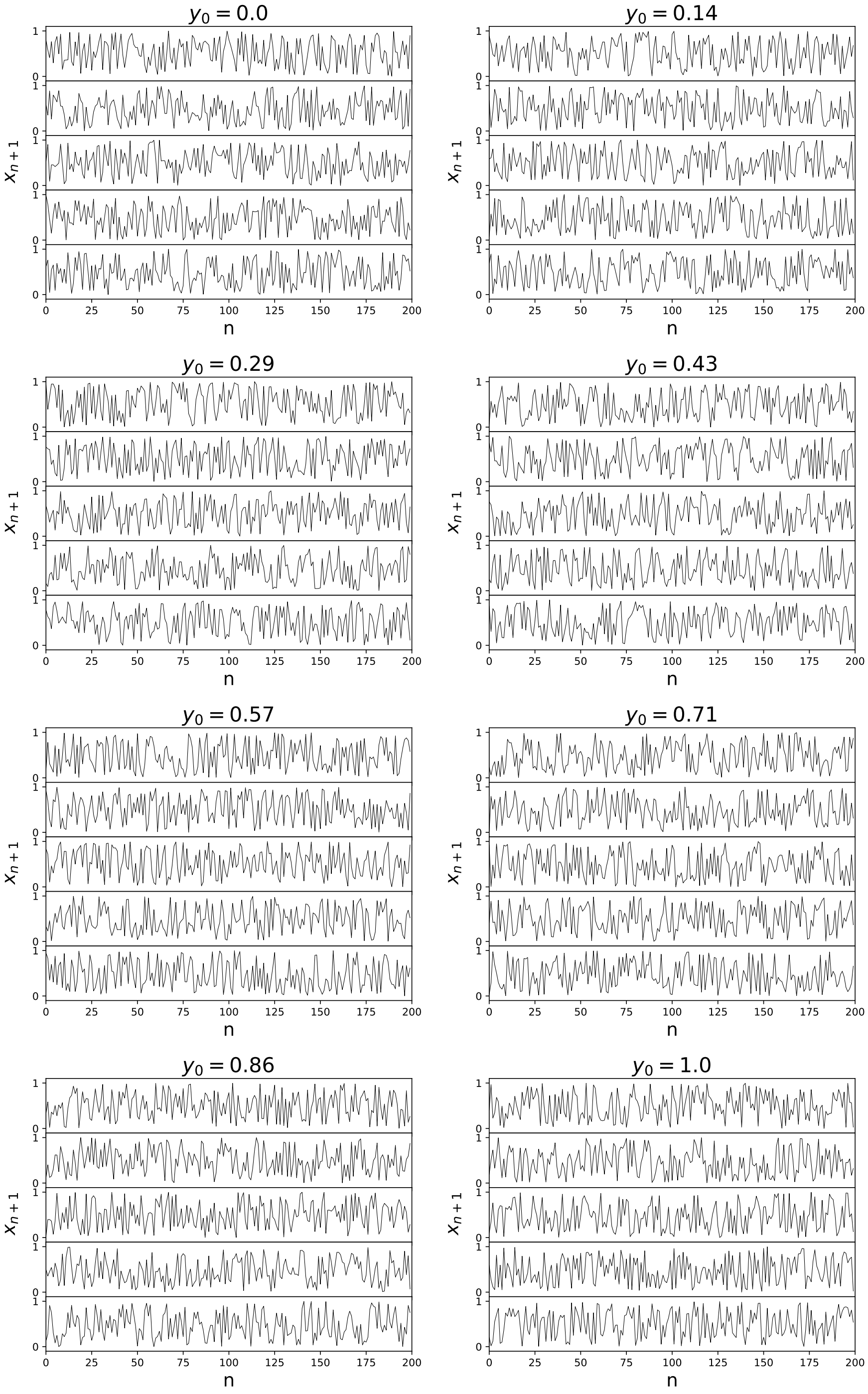

Figure S5: The time-dependent Zaslavskii map with selected y_0 values and $v = 400, a = 12, r = 3$. Each subplot shows 5 sequences (top to bottom) with $x_0 = 0.1, 0.3, 0.5, 0.7$, and 0.9, respectively.

II. Virtual screening results of the DUD-E nuclear receptor subset

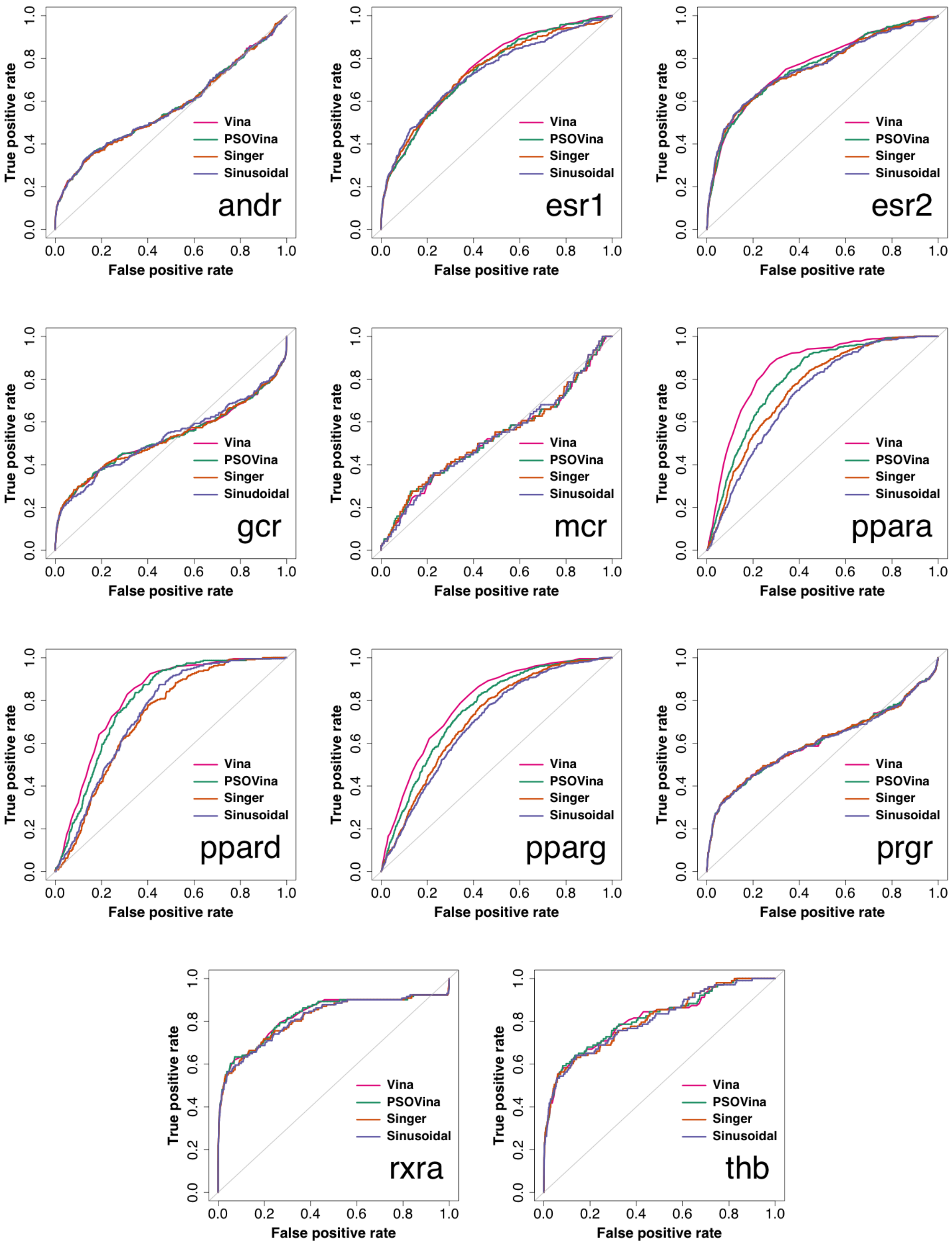

Figure S6: ROC curves of virtual screening the DUD-E nuclear receptor targets (NR11) using AutoDock Vina, PSOVina, and chaos-embedded PSOVina^{2LS} with Singer and sinudoidal maps.

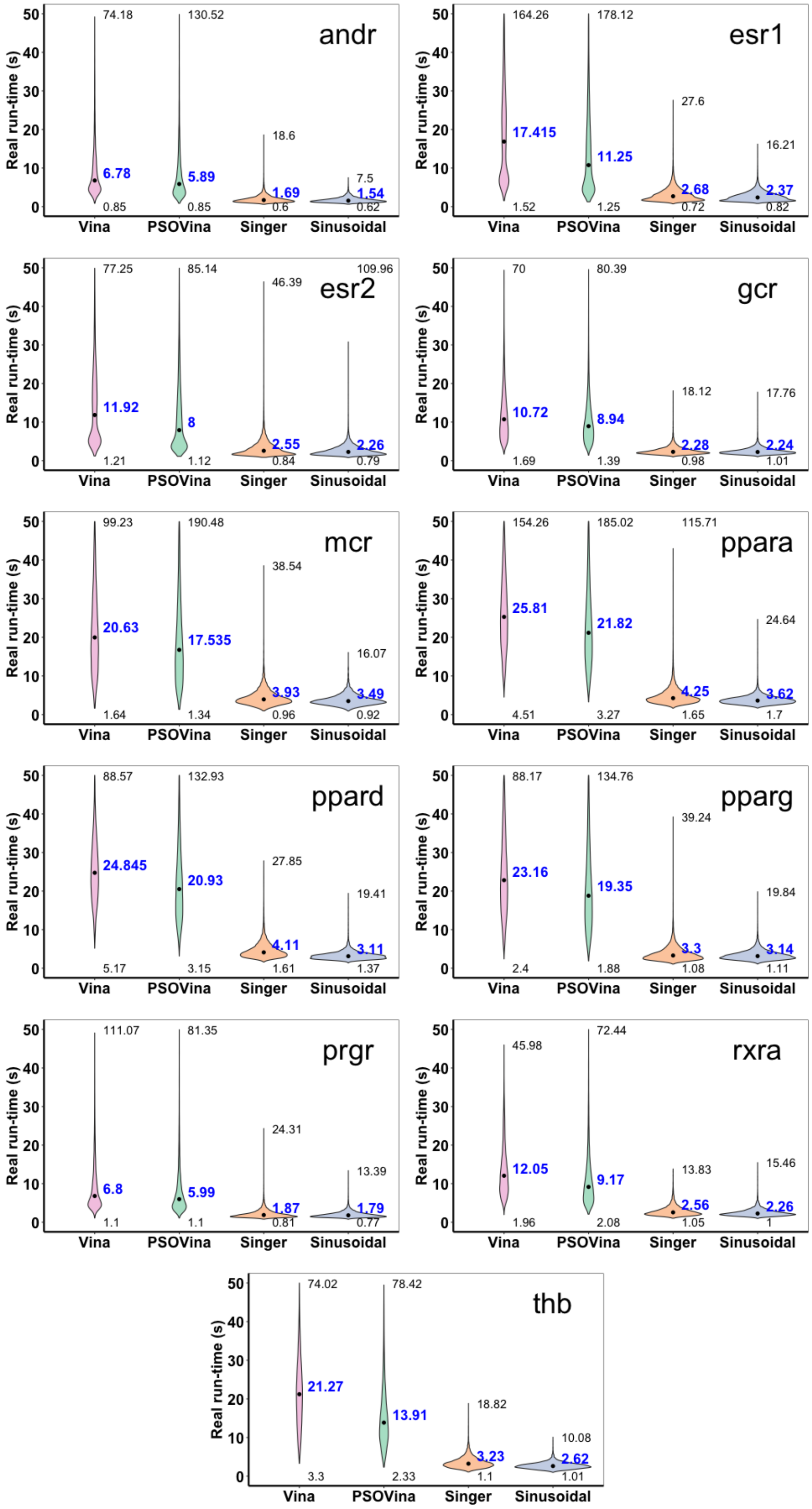

Figure S7: Run time (in seconds) of virtual screening the DUD-E nuclear receptor targets (NR11). Text annotations in the violin plot indicate the maximum (top), median (text in blue, location of the median shown as a black dot), and minimum (bottom) run times by each method.

III. Statistical test results of virtual screening performances

Table S1: P-values of virtual screening accuracy between pairs of the docking methods by Student's t-test.

(a) DIV8: p-values for AUC-ROC

	PSOVina	Singer	Sinusoidal
AutoDock Vina	0.502239628	0.658508424	0.711338294
PSOVina		0.860759872	0.995441826
Singer			0.672577076

(b) DIV8: p-values for $EF_{1\%}$

	PSOVina	Singer	Sinusoidal
AutoDock Vina	0.208613107	0.094591074	0.789945513
PSOVina		0.578061881	0.739105529
Singer			0.532203606

(c) DIV8: p-values for $EF_{20\%}$

	PSOVina	Singer	Sinusoidal
AutoDock Vina	0.653235697	0.28587446	0.270771807
PSOVina		0.209034991	0.208590894
Singer			0.699327083

(d) NR11: p-values for AUC-ROC

	PSOVina	Singer	Sinusoidal
AutoDock Vina	0.074559343	0.052079418	0.049957785*
PSOVina		0.062446538	0.042898042*
Singer			0.343681062

(e) NR11: p-values for $EF_{1\%}$

	PSOVina	Singer	Sinusoidal
AutoDock Vina	0.020254118*	0.206574478	0.172905418
PSOVina		0.780670372	0.970049433
Singer			0.69139361

(f) NR11: p-values for $EF_{20\%}$

	PSOVina	Singer	Sinusoidal
AutoDock Vina	0.032844353*	0.041994057*	0.047549751*
PSOVina		0.074916477	0.076160622
Singer			0.470099755

**,* significant at 1% and 5%, respectively

Table S2: P-values of virtual screening run time performance between pairs of the docking methods by Student's t-test.

(a) DIV8: p-values for run time

	PSOVina	Singer	Sinusoidal
AutoDock Vina	0.056566288	0.000852785**	0.000911575**
PSOVina		0.000267703**	0.000326166**
Singer			0.030963625*

(b) NR11: p-values for run time

	PSOVina	Singer	Sinusoidal
AutoDock Vina	0.000177163**	0.000030551**	0.000032386**
PSOVina		0.000075421**	0.000076910**
Singer			0.018167528*

**,* significant at 1% and 5%, respectively
